# Controllable AgNPs encapsulation to construct biocompatible and antibacterial titanium implant

**DOI:** 10.3389/fbioe.2022.1056419

**Published:** 2022-11-30

**Authors:** Zhangao Wei, Kexin Li, Shuang Wang, Lan Wen, Linghan Xu, Yankai Wang, Zirui Chen, Wei Li, Hua Qiu, Xiangyang Li, Jialong Chen

**Affiliations:** ^1^ Key Laboratory of Oral Diseases Research of Anhui Province, Stomatologic Hospital and College, Anhui Medical University, Hefei, China; ^2^ Department of Neurosurgery, West China Hospital, Sichuan University, Chengdu, China

**Keywords:** AgNPs, pDA shell, one-pot method, biocompatible, anti-bacterial

## Abstract

Silver nanoparticles (AgNPs) are progressively becoming an in-demand material for both medical and life use due to their effective antimicrobial properties. The high surface area-to-volume ratio endows AgNPs with enhanced antibacterial capacity accompanied by inevitable cytotoxicity. Surface coating technique could precisely regulate the particle shape, aggregation, and Ag^+^ release pattern of AgNPs, by which the cytotoxicity could be significantly reduced. Various coating methods have been explored to shell AgNPs, but it remains a great challenge to precisely control the aggregation state of AgNPs and their shell thickness. Herein, we proposed a simple method to prepare a tunable polydopamine (pDA) coating shell on AgNPs just by tuning the reaction pH and temperature, yet we obtained high antibacterial property and excellent biocompatibility. SEM and TEM revealed that pDA coated AgNPs can form core-shell structures with different aggregation states and shell thickness. Both *in vitro* and *in vivo* antibacterial tests show that acid condition and heat-treatment lead to appropriate AgNPs cores and pDA shell structures, which endow Ti with sustained antibacterial properties and preferable cell compatibility. One month of implantation in an infected animal model demonstrated that the obtained surface could promote osteogenesis and inhibit inflammation due to its strong antibacterial properties. Therefore, this study provides a promising approach to fabricate biocompatible antibacterial surface.

## Graphical Abstract

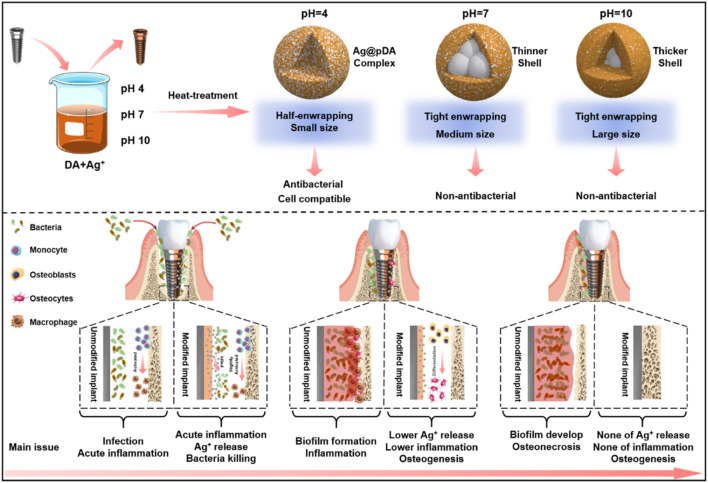

## Introduction

The number of orthopedic/dental implant failures increases year by year, primarily due to bacterial infection; accordingly, antibacterial materials that prevent bacterial infections have attracted increasing attention (Wang et al., 2021a). Various strategies for fabricating antibacterial surfaces on titanium have been developed (Elliott et al., 2021; Ghilini et al., 2021). However, such surfaces are used as bone-related antibacterial materials, the long-term stability and biosafety are considered the two most important properties in this field. Surface modification to endow titanium with the aforementioned properties is the main method to constructing ideal orthopedic/dental implants (Yang et al., 2017; Kitagawa et al., 2021). To date, various antibacterial agents have been developed for the surface modification of implants, including antibiotics (Hasan et al., 2021), antimicrobial peptides (Wu et al., 2021), and antibacterial metallic elements (Zhang et al., 2016). Among these agents, silver is an enduring research hotspot for antibacterial surface preparation due to its broad-spectrum effectiveness, low resistance and high efficiency (Prencipe et al., 2021). However, considering the cytotoxicity of silver, ideal silver-modified surfaces must be able to balance antibacterial behavior and osteogenic response in accordance with the dose and loading behavior (Pryshchepa et al., 2020).

The cytotoxicity and antibacterial ability of silver nanoparticles (AgNPs) are both strongly dependent on the amount, size, shape, loading behavior and other physiological characteristics. AgNPs showed a clear effect on lactate dehydrogenase activity, reactive oxygen species (ROS) generation and DNA damage in a size-dependent manner (Akter et al., 2018; Yaqoob et al., 2020). Wang et al. reported that AgNPs with a size of 20 nm showed more cytotoxicity than 110 nm AgNPs, and further investigation indicated that smaller AgNPs cause acute neutrophilic inflammation in mouse lung (Wang et al., 2014). The influence of particle size may be ascribed to the uptake mechanism of Ag, which will ultimately affect its cytotoxicity. Coating is a conventional strategy to modulate the release of Ag^+^ and produce electrostatic repulsion, thus helping stabilize the particles (Guo et al., 2016; Fahmy et al., 2019). Uncoated AgNPs kill cells in a size- and dose-dependent manner, while coating can affect the shape, aggregation, and surface electrical behavior of AgNPs, which prevents direct toxicity. Various coatings, including organic agents (polyvinylpyrrolidone (Kyrychenko et al., 2015), citrates (Gutierrez et al., 2015), proteins (Chakraborty et al., 2018) and inorganic agents (sulfide, chloride and carbonate), have been developed to modify AgNPs. As reported, citrate- and polyvinylpyrrolidone-coated AgNPs showed increased stability and reduced cytotoxicity compared with bare AgNPs (Nguyen et al., 2013). However, both organic and inorganic coatings are characterized by the disadvantages of complex processes, high cost and poor controllability.

Mussel-inspired surface modification has attracted extensive attention since it was reported by Haeshin Lee et al. (2007). Dopamine, a mussel-inspired molecule, was reported to form a material-independent coating based on covalent bonding and π–π stacking through oxidative polymerization and noncovalent bonds (Saiz-Poseu et al., 2019). Subsequently, they proposed that catechol/o-quinone can trigger strong coordination with metals and reduce noble metal ions to form metal NPs on diverse surfaces (Hong et al., 2011). Thus, mussel-inspired chemistry started a new chapter in the field of AgNPs, and various studies on polydopamine (pDA)-mediated AgNPs have been carried out (Jo et al., 2014; Islam et al., 2018). First, researchers mainly focused on the formation of AgNPs with strong antibacterial properties, but with the deepening of research, more attention is being given to the balance of biosafety and biofunction (Alarcon et al., 2012; Yang et al., 2020). Changsheng Zhao et al. fabricated AgNP-loaded carbon nanotubes by means of dopamine-mediated chemistry, and both gram-negative and gram-positive bacteria were effectively killed. However, significant negative effects were observed in *in vitro* cell culture due to the cytotoxicity of Ag. To improve compatibility, they used heparin and chitosan to reduce the amount of Ag and adjust its loading behavior which ultimately resulted in an antibacterial material with good cell compatibility (Nie et al., 2016). While these previous researchers favored the use of additives to control the shape and release mechanism of AgNPs, they overlooked the fact that dopamine is a first-class coating material that can be successfully wrapped around the target under proper reaction conditions. Jiuju et al. used a similar one-step method to prepare AgNPs@PDA composites in the field of photocatalysis. The particles had core-shell structure, which gave us great encouragement, that is, the simple preparation method could make dopamine wrapped silver particles (Feng et al., 2012). Using polydopamine as an adhesive, reducing agent, diffusion barrier and inducer, Yan Cheng et al. constructed nanocomposite Ag/Cap coatings on titanium dioxide nanotubes under proper reaction conditions, which enabled TiO2 surface to have osteointegration-promoting and antibacterial properties without requiring complex equipment and reaction steps (Li et al., 2015).

Generally, pDA coatings are prepared in alkaline environments because alkaline conditions benefit the oxidative polymerization of catecholamines, which promotes pDA coating formation (Bernsmann et al., 2011). However, many researchers overlooked the fact that in acidic environments, the stability of the transient oxidation intermediates of catechol will increase, resulting in a decreased self-polymerization rate (Zheng et al., 2015), which may favor the enwrapping of AgNPs. Moreover, metal ions with strong oxidizability can accelerate oxidative polymerization even under acidic conditions (pH = 4). David Ruch et al. proposed that oxidative metal ions can receive the electrons produced in the oxidation of dopamine, which will promote polymerization (Ball et al., 2013). Furthermore, ion-dopamine coordination may enable linking between the intermediate state of pDA produced in solution and surfaces and hence promote the formation of stable coatings (Ejima et al., 2017; Wang et al., 2020). Therefore, different pH values will cause different polymerization forms of pDA, hence leading to different degrees of reduction and enwrapping of AgNPs, which will open the door for fabricating controllable antibacterial pDA@AgNP surfaces.

Herein, we developed a novel one-pot method to construct dopamine/silver composite antibacterial surfaces. pH and temperature are two variables in the reaction system. Different pH values were set to adjust the oxidation degree of dopamine; variations in pH will also influence the participation degree of Ag^+^ in the redox/coordination reaction between Ag^+^ and dopamine, which will ultimately determine the enwrapping degree of the resultant pDA@AgNP surface. Heat treatment after simple cleaning was performed as a crucial procedure that will recapture residual dopamine and initiate further oxidation crosslinking (Luo et al., 2013), thus forming a stable coating surrounding the AgNPs to slow Ag release and reduce cytotoxicity. The results indicate that the dopamine/Ag composite surface prepared under acidic conditions had the best antibacterial properties. Heat treatment increased the durability of the surface antibacterial ability and reduced cytotoxicity, which has applicable value for preventing implant infection complications.

## Materials and methods

### Materials

Commercially pure titanium was purchased from Baoji Nonferrous Metal Co., Ltd. Dopamine-HCl, silver nitrate, rhodamine 123 and MTT were purchased from Sigma-Aldrich. Nitric acid, hydrochloric acid, dimethyl sulfoxide and glutaraldehyde were purchased from Acros Organics. Fetal bovine serum, α-minimum Eagle’s medium, and trypsin-EDTA solution were purchased from Gibco. Other reagents were purchased from Sinopharm Chemical Reagent Co., Ltd.

### Preparation of Ag/dopamine coating

After being mechanically polished to 2000 grit, the titanium discs were ultrasonically cleaned successively with acetone, ethanol and deionized water and denoted Ti. Then, Ti was immersed in a 2.5 M NaOH solution at 60°C for 24 h and was ultrasonically cleaned in deionized water at 100°C for 2 h, allowing it to acquire the micro/nanoporous structure, which was denoted as pTi. After that, the sample was immersed in a mixed solution of dopamine (2 mg/mL) and silver nitrate (0.02 mg/mL) at three pH values (4, 7, and 10). After reaction for 24 h at 37°C in a light-free environment, all the samples were removed, and each group was then divided into two identical groups. Half of the samples in each group were cleaned by ultrasound in deionized water for 3 min and were denoted as pH 4, pH 7 and pH 10. The other half were transferred to the oven for 2 h at 150°C, cleaned by ultrasound in deionized water for 3 min, and denoted as pH 4-H, pH 7-H and pH 10-H.

### Surface characterization

The surface morphology and structure of the samples were investigated using scanning electron microscopy (SEM) with an accelerating voltage of 3 kV, and the distribution of silver on the surfaces and solutions was investigated using detectors for backscatter electron images (BSE) with an accelerating voltage of 10 kV and transmission electron microscope (Hitachi, Tokyo, Japan), respectively. X-ray photoelectron spectroscopy (XPS) with an Al Ka X-ray source (1,486.6 eV photons) was applied to analyze the surface chemical composition. A survey scan was performed between 0 and 1,100 eV electron binding energies at a pass energy of 100 eV, and a high-resolution spectral scan was performed at 30 eV to obtain detailed information. Due to the 45° take-off angle in XPS, the maximum detection depth did not exceed 10 nm. To investigate the total amount of silver loaded on the coating, the samples were immersed in 2 mL aqua regia for 2 h and were then diluted with deionized water to 10 mL to measure the silver concentration by inductively coupled plasma-mass spectrometry (ICP-MS). To study the release behavior of silver in coatings, the samples were immersed in 10 mL physiological saline at 37°C. At a scheduled time (1, 3, 5, 7, 14 days), the entire volume was collected, and fresh physiological saline was added. The silver concentration in the collection solution was measured by ICP-MS.

### *In Vitro* antibacterial test experiments

During implantation, bacteria in the mouth can attach to implant surfaces or invade implant sites through the crevice between implants and surrounding tissues, leading to infection or inflammation (peri-implant inflammation and peri-implant mucositis). Therefore, antibacterial implants should inhibit bacterial adhesion and biofilm formation on the implant surface and inhibit the bacteria in surrounding tissues to avoid infection-associated inflammation. Thus, the evaluation of antibacterial ability of materials involves two parts. One is to evaluate the inhibitory ability of samples to bacteria in the surrounding environment by the turbidimetric method, inhibition ring method and serial dilution method, and the other is to evaluate the ability of sample surface to inhibit bacterial adhesion and biofilm formation by live/dead bacteria staining and the spread plate method. Here, *Staphylococcus aureus* (*S. aureus*) was incubated aerobically in brain heart infusion (BHI) broth supplemented with 2% sucrose. *Actinobacillus actinomycetemcomitans (Aa)* was incubated anaerobically (85% N_2_, 10% H_2_, 5% CO_2_) on horse blood agar. Then, a colony was removed and added to Luria−Bertani (LB) broth and diluted to 10^5^ CFU/mL to obtain the test strain solution for the following antibacterial evaluation. 1) Turbidimetric method: 60 μl aliquots of test strain solution were distributed onto the sample surfaces in a 24-well plate, and after incubation for 4 h at 37°C, 2 mL of LB broth was added to each well and cultured at 37°C. After incubation for 24 h with *S. aureus* and 48 h with *Aa*, 100 μl of LB broth was removed, and the optical density was measured at 660 nm (OD660) using a microplate reader. 2) Inhibition ring method: 100 μl of *S. aureus* or *Aa* test strain solution was spread evenly onto a solid LB agar or a horse blood agar plate surface, and then the samples were lightly placed face down on the solid agar. After an incubation of 24 h for *S. aureus* and 48 h for *Aa* at 37°C, the transparent inhibition zone around the sample was photographed. 3) Serial dilution method: test strain solutions of *S. aureus* with different dilutions were incubated with samples for 1, 3, and 5 days, and the turbidity of the solution was photographed. 4) Live/Dead bacteria staining and spread plate method: 60 μl aliquots of the test strain solution were distributed onto the sample surfaces in a 24-well plate, and after incubation for 4 h at 37°C, 2 mL of LB broth was added to each well and cultured at 37°C. After incubation for 24 h with *S. aureus* and 48 h with *Aa*, the samples were removed, rinsed with physiological saline and stained using a LIVE/DEAD Bac Light Bacterial Viability Kit. In addition, the samples were put into a sterilized centrifuge tube with 2 mL of physiological saline. After ultrasonic treatment for 2 min and vortexing for 5 min and 1000-fold dilution, 100 μl aliquots with *S. aureus* or *Aa* were removed and spread evenly onto a solid LB agar or horse blood agar plate surface, respectively. After an incubation of 24 h for *S. aureus* and 48 h for *Aa*, the CFUs on the plates were observed.

To evaluate the stability of the antibacterial ability of the samples, multiple inoculations with different dilutions of *S. aureus* were used to simulate the continuous invasion of bacteria in the implant site, and the samples were immersed in physiological saline for 7 days. Then, the samples were evaluated for antibacterial performance by the turbidimetric method and spread plate method with S. aureus at 10^5^ CFU/mL.

### Cytocompatibility evaluation

The mouse osteoblastic cell line (MC3T3-E1) and the mouse fibroblastic cell line (NIH-3T3) were employed to assess the cytotoxicity. Cells were cultured in α-MEM containing 15% fetal bovine serum in a humidified atmosphere of 5% CO_2_ at 37°C. After UV sterilization, the samples were placed in a 24-well plate. A 1 mL cell suspension with a density of 5 × 10^4^ cells/mL was added to each well, and the medium was refreshed every 2 days. After incubation for 1, 3, and 5 days, the samples were rinsed with sterile physiological saline, and some samples were transferred to 2.5% glutaraldehyde and underwent a 4 h for fixation. Then, the samples were stained with YF555-phalloidin for F-actin and DAPI for nuclei, and the other samples were used for MTT tests. In addition, to assess the toxicity of the material to the surrounding tissue, a 1 day extraction of the samples were collected to assess their toxicity by crystal violet staining and MTT tests. The percent inhibition was calculated by the following formula:

relative growth rate(RGR)(%)=(OD490 nm value of sample well−OD490 nm value of blank well)/(OD490 nm value of control well−OD490 nm value of blank well)×100

### Competitive bacterial-cell adhesion

First, 60 μl of LB broth containing *S. aureus* or *Aa* at a density of 10^5^ CFU/mL was distributed onto sample surfaces and incubated for 4 h at 37°C. Then, 1 mL of cell suspension (MC3T3-E1 or NIH-3T3) with a density of 5 × 10^4^ cells/mL was inoculated onto the samples. After incubation for 1 and 3 days at 37°C and 5% CO_2_, the samples were rinsed with physiological saline. Then, both the live and dead bacteria/cells on the samples were stained using the LIVE/DEAD Bac Light Bacterial Viability Kit to assess competitive bacterial-cell adhesion to the different materials.

### *In Vivo* experiments

After anaesthetization by intraperitoneal injection of 1% pentobarbital, both hind legs of SD rats were shaved, depilated, and disinfected with iodine. Then, the femoral condyles of both hind legs were exposed by skin incision, and a Kirschner wire was employed to drill a hole through the cortical and cancellous bones. At the same time, a 10 mm longitudinal skin area was cut to expose the superficial layer of the deep fascia to form a pocket. Sterile titanium rods (2 mm in diameter and 8 mm in length) or titanium discs (1 cm in diameter and 1 mm in thickness) with different surface modifications were immersed into the *S. aureus* suspensions at a concentration of 10^5^ CFU/mL for 1 h, and then the samples were inserted in the legs or pockets in the skin. After implantation, the hole was blocked by bone wax, and the wound in the leg and skin was closed with a suture. After 1 and 7 days, some of the samples were explanted, rolled on solid LB agar plates and recultured for 1 days. In addition, other samples were immersed in 1 mL of physiological saline with ultrasonic and vortex treatment, and then 100 μl aliquots were removed and spread evenly onto solid LB agar plates. The samples were recultured for 1 days, and the CFUs on the plates were observed. After 1 month, the animals were euthanized, and the titanium rods and discs with the surrounding tissues were harvested for further micro-CT analysis and histological analysis, respectively.

### Statistics

All experiments were performed at least three independent times. All quantitative data are presented as the means ± standard deviations and were compared with one-way ANOVA tests to evaluate statistical significance using SPSS software. After ANOVA, Tukey’s multiple comparisons test was performed to find significant differences between pairs.

## Results and discussion

### Surface morphology of AgNPs-loaded coatings

**FIGURE 1:**
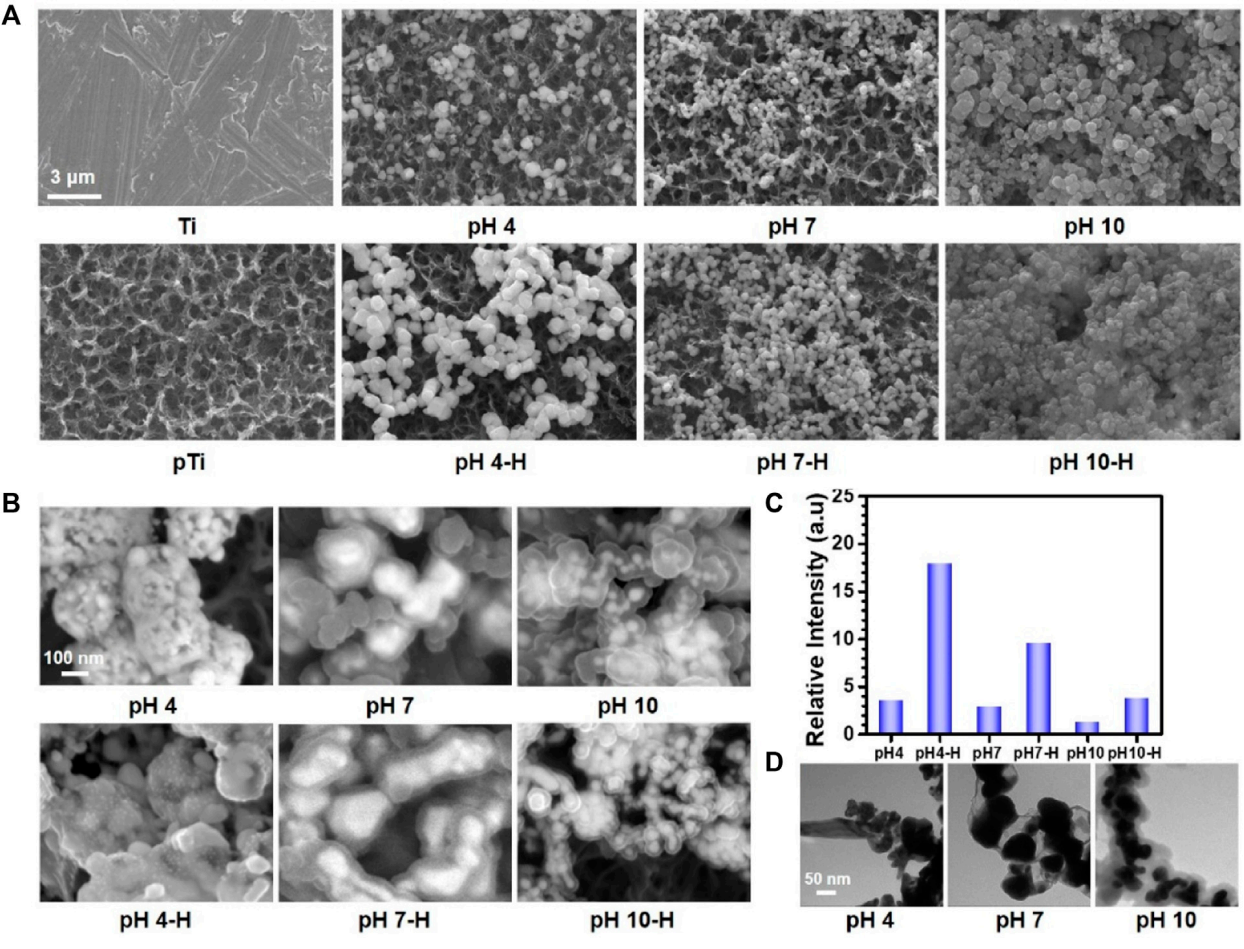
The surface morphology of different samples: **(A)** scanning electron microscopy (SEM) images of different samples; **(B)** Backscattered electron (BSE) images of different samples; **(C)** relative intensive of Ag by X-ray photoelectron spectroscopy (XPS); **(D)** Transmission electron microscope (TEM) analysis of AgNPs in solutions at pH 4, pH7, pH10.

As the scanning electron microscopy (SEM) images in Figure 1A showed, after alkali-heat treatment, the Ti surface presented a uniform micro/nanoporous morphology with increased specific surface area. After immersion in the dopamine/silver solution, a large number of AgNPs were coupled with the porous surfaces. In addition, with increasing pH, the number of AgNPs increased, and their size decreased; as a result, the pH 10 surface was completely covered by AgNPs (Fernando and Zhou, 2019). Compared with the unheated surfaces, the number of AgNPs significantly increased on the heated surfaces, and the AgNPs tended to merge together with the aid of residual dopamine which more closely resembled a pDA@NPs complex than two separate parts. To further study the loading behavior of AgNPs in the pDA@NPs complex, backscattered electron (BSE) imaging was performed. Backscattered electrons are high-energy electrons that can be used to obtain high-resolution images of the distribution of elements in a sample (Maitland and Sitzman, 2007). As displayed in Figure 1B, the complexes at pH 7 and pH 10 showed an obvious change in which the highlighted core was covered by a translucent shell, which may indicate that Ag was warpped by pDA. Of particular interest was the enwrapping manner of Ag in pDA@NP complexes prepared at different pH values. Under neutral and alkaline conditions (pH = 10), Ag cores were separately coated by shells, while in acidic conditions (pH = 4), many Ag cores were assembled first and then coated with pDA. Since alkaline conditions can accelerate the oxidative polymerization of dopamine (Saiz-Poseu et al., 2019), we speculate that increased dopamine cross-linking led to high shell shrinkage and the accelerated oxidative polymerization of dopamine favored the enwrapping of silver particles. However, under acidic conditions, the driving force of dopamine oxidative polymerization was mainly provided by Ag^+^, so the polymerization was slower. In addition, pH value also affected the size of Ag core, which gradually decreases with the increase of pH value, which provides favorable conditions for dopamine encapsulation (Fernando and Zhou, 2019; Marciniak et al., 2020). And the two processes of Ag core formation and enwrapping proceeded together, which may led to this specific coating.

In contrast to the thickly wrapped samples, AgNPs at pH 4 showed tight but not very thick enwrapping, which was also demonstrated in the X-ray photoelectron spectroscopy (XPS) results. As Figure 1C shows, after heat treatment, the amount of Ag increased significantly, and this effect was most pronounced at pH 4. Since the detection depth of XPS is approximately 10 nm, atoms deeper than 10 nm cannot be effectively detected (Guy and Walker, 2016). Therefore, even the pDA@NP complex displayed in Figure 1A did not show much of a difference between pH 4-H, pH 7-H and pH 10-H, but the atomic percent of Ag exhibited a downward trend with increasing pH (Figure 1C). To further explore the Ag distribution at different pH values, transmission electron microscope (TEM) analysis was carried out (Padhi and Behera, 2022). As the results in Figure 1D showed, high-density and low-density substances were observed, which correspond to Ag and pDA, respectively. At pH 4, AgNPs were closely wrapped by pDA, and some AgNPs were exposed in the solution, which may benefit the release and function of Ag. In contrast, in the particles prepared at pH 7 and 10, Ag was coated by a greater thickness of pDA than that at pH 4, which is consistent with the results of BSE and XPS analysis. In conclusion, pH 7 and more alkaline conditions will accelerate the polymerization of dopamine and consequently form a thicker coating outside the AgNPs, which may influence the release mechanism and function of AgNPs.

### Characterization of the pDA@NPs complex

**FIGURE 2:**
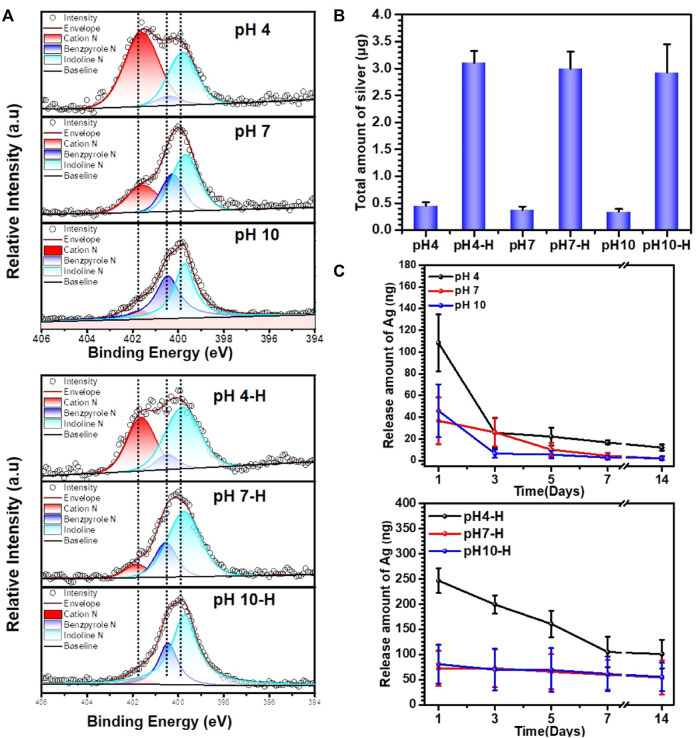
Characterization of different samples: **(A)** XPS high-resolution N analyses of pH4-H, pH7-H and pH10-H; **(B)** total amount of Ag in different samples investigated by Inductively Coupled Plasma Mass Spectrometry (ICP-MS); **(C)** the release amount with time of different samples detected by ICP-MS.

To further analyze the chemical composition and structure of the pDA@NPs complex, XPS full-spectrum and high-resolution N analyses were carried out. As the results in Supplementary Figure S4 show, with the formation of the pDA@NPs complex, the N1s peak increased, and the Ag3d peak appeared. Since the detection depth of XPS was limited, the Ti2p peaks significantly decreased after samples were coated by the pDA@NP complex (Guy and Walker, 2016). As the pH value of the mixed solution increased, the intensity of the Ag3d and Ti2p peaks decreased regardless of the use of heating, demonstrating that more AgNPs were formed in acidic conditions, which was consistent with the BSE results. Moreover, the intensity of the Ag3d peaks on the heated surfaces was significantly higher than that on the unheated surfaces because there must be Ag^+^ and dopamine in the residual solution, and heat treatment will trigger further crosslinking of dopamine and Ag^+^, which ultimately cause the relocation of the pDA@NPs complex (Zhang et al., 2016). Moreover, heating made the coating more stable, so more particles were retained after ultrasonic cleaning, which was in agreement with the results of the surface morphology tests. To extensively analyze the status of the pDA@NPs complex, high-resolution N spectra were collected. As displayed in Figure 2A, the dopamine molecule can be oxidized in the presence of oxygen in solution, followed by the formation of an indoline moiety through Michael addition (Saiz-Poseu et al., 2019). Then, the molecule undergoes further oxidation and is rearranged to a benzopyrrole-type molecule, a more stable form of oxidized dopamine. During this process, two kinds of nitrogen, indoline N and benzopyrrole N, formed (Liebscher et al., 2013). Ammonium ion (cation N) also existed in the pDA coating due to the protonation of N-containing groups. Based on the understanding of the resulting structure of oxidized dopamine, the peaks at 399.8, 400.4, and 401.6 eV can be assigned to indoline N, benzopyrrole N and cation N according to the charge of N atom.

At lower pH, the ionization of phenol is inhibited, while the ionization of amino groups is enhanced, which leads to a reduced polymerization rate of dopamine and the formation of many cation N groups in the pDA coating (Liu et al., 2014). The oxidation degree of dopamine will increase in alkaline solution, while the content of cation N will decrease significantly through a possible deprotonation process. Moreover, indoline N and benzopyrrole N also tended to increase. These results indicated that, consistent with previous reports, a higher pH contributes to the deep oxidation of dopamine (Vatrál et al., 2015; Wang et al., 2019).

The intragroup variation in N contents in the high-temperature-treatment group under different pH values was similar to that in the room-temperature-treatment group. According to intergroup comparison, the high-temperature treatment led to a dramatic decrease in cation N at a certain pH. However, the changes in the contents of indoline N and benzopyrrole N were different. It is generally believed that oxidation will lead to more benzopyrrole N under high-temperature treatment, but the data show that the benzopyrrole N content decreased instead (Lee et al., 2007). One possible interpretation is that indoles are reduced to an indoline structure by silver at high temperature.

As demonstrated above, pH and heat treatment play vital role in the pDA@NP complex formation, which may influence the Ag^+^ release mechanism (Zheng et al., 2015). To quantify the total amount of Ag and detect the Ag release mechanism, Inductively Coupled Plasma Mass Spectrometry (ICP-MS) was used. As shown in Figure 2B, pH did not influence the volume loading of Ag, but heat treatment significantly improved it. After simple cleaning, a certain amount of Ag and dopamine was retained. During heat treatment in air, the oxidative polymerization triggered by both oxygen and Ag^+^ increase tremendously, which results in a higher loading capacity of Ag and a specific coating manner (Proks et al., 2013). Accordingly, the release behavior was correlated with the loading method to a certain degree. As shown in Figure 2C, different pH values did not substantially influence the release of Ag when temperature was not considered. However, in the heat-treatment groups, the release amount of Ag increased with an elevated loading amount, among which the initial release of pH 4-H reached 250 ng on the first day, and a release plateau of 100 ng was maintained at a prolonged detection time of 7–14 days.

In conclusion, the fine spectra of N1s (Figure 2A) showed that the degree of dopamine oxidative polymerization was enhanced with increasing pH, SEM results showed that the coating thickness on titanium sheet gradually increased with the increase of pH (Figure 1A). ICP-MS showed that there was no evident distinction in the total amount of silver in each group (Figure 2B). It indicated that the amount of substance increased in the coating was mainly formed by oxidative polymerization of dopamine into a polymer and then precipitation at high pH. For silver, silver nitrate would form sediment under both acidic and alkaline conditions, so pH had little effect on the amount of silver deposited, as shown by ICP-MS. In addition, the higher the pH was, the faster the oxidative polymerization reaction of dopamine. When silver had not formed a large core, it was wrapped to isolate its reaction. Moreover, the higher the pH was, the faster the oxidative polymerization reaction would be and the deeper the degree would be, so the thicker the dopamine shell would be. As shown in the results of BSE (Figure 1B) and TEM (Figure 1D), the higher the pH was, the thicker the dopamine shell and the smaller the silver core. Therefore, even if the amount of substance in the coating increased under high pH conditions, the increased component was dopamine, and the proportion of silver in the coating decreased and could not be released by wrapping. Therefore, the relative strength results of surface elements in XPS showed that the strength of silver gradually decreased because the measured depth and surface area were constant (Figure 1C, Supplementary Figure S4). In addition, when the pH was lower, silver would also precipitate and polymerize, but at this time, the degree of oxidative polymerization of dopamine was low and mainly depended on the reaction and cross-linking polymerization with silver. Therefore, at pH4, the thickness of the coating was significantly reduced, and the main component of the coating was silver. Because dopamine was polymerized mainly by cross-linking with silver, the particles formed by the reaction looked like complex particles rather than inclusions. By heating the sample, the loading of the coating was mainly improved, and the reaction of dopamine and silver was not significantly affected. In summary, the innovative AgNPs preparation system reused residual dopamine and Ag^+^ and significantly increased the loading of Ag; heat treatment improved the oxidative crosslinking of dopamine, which enhanced the stability of the pDA@Ag complex; and pH played a key role in determining the Ag enwrapping method, which ultimately led to sustained and smooth release.

### *In Vitro* antibacterial properties

**FIGURE 3:**
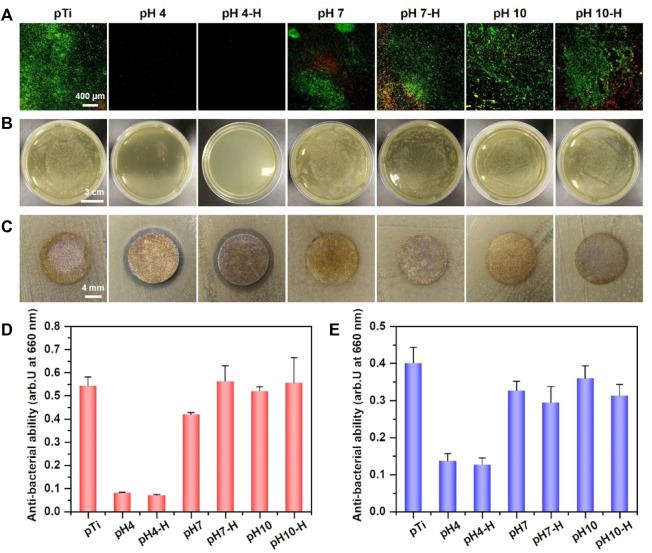
Assessment of antibacterial activity of different samples: **(A)** Live/Dead bacteria staining of S. aureus on different samples; **(B)** antibacterial property (S. aureus) of various sample surfaces determined by spread plate method; **(C)** the diameter of inhibition zone around various samples for S. aureus; **(D)** bacterial amount in the inoculum after incubation of various samples with S. aureus for 24 h by turbidimetric method; **(E)** bacterial amount in the inoculum of various samples after incubation with Aa for 24 h by turbidimetric method.

Antibacterial surface modification endows implants with appropriate antibacterial properties to inhibit bacterial adhesion and biofilm formation after implantation. Fürst et al. (2007) reported that bacterial colonization occurred within 30 min after implantation, among which *S. aureus* and *Aa* were the main species that contributed to local implant infection, even in the oral cavity. Here, live/dead bacteria staining and the spread plate method were used to evaluate the ability of the prepared surface to inhibit bacterial adhesion or kill adhered bacteria. First, *in situ* evaluation of adhered *S. aureus* and *Aa* was performed by fluorescent staining of live/dead bacteria. As shown in Figure 3A and Supplementary Figure S1A, many bacteria adhered rapidly to the surface of pTi, and most of the adhered bacterial cells were alive (stained green). Among the pDA@NP-modified surfaces, only the pH 4 and pH 4-H surfaces completely inhibited bacterial adhesion. Moreover, the spread plate method was used to observe the number of live *S. aureus* and *Aa* adhered to the surface. As shown in Figure 3B and Supplementary Figure S1B, there was almost nothing on the surface of pH 4-H, and sporadic live bacteria were observed on pH4. In contrast, many live bacteria adhered to the dopamine/Ag-modified surfaces prepared in neutral and alkaline conditions, which was attributed to the distribution of silver on the surface: the dopamine shell made it difficult for silver to directly contact bacteria and exert antibacterial effects. These results show that the surfaces of pH 4 and pH 4-H samples possess the best antibacterial ability, which is consistent with the ICP-MS and XPS results that acid conditions led to the formation of more pDA@NP complexes and a better Ag release process.

To further evaluate the antibacterial properties of the surroundings of these samples, the inhibition zone method (Figure 3C and Supplementary Figure S1C), turbidimetric method (Figure 3D) and serial dilution method (Supplementary Figures S2A–C) were carried out. After incubation with bacterial suspensions (*S. aureus* and *Aa*) for 24 h, the optical density at 660 nm (OD 660) was measured to evaluate the indirect antibacterial ability of different samples. As shown in Figure 3D (*S. aureus)* and Figure 3E *(Aa*), only pH 4 and pH 4-H exhibited better antibacterial activity. The results of the zone of inhibition were the same as those of the turbidimetric method (Figure 3C and Supplementary Figure S1C): the only clear inhibition zone was observed around the pH 4 and pH 4-H samples. In addition to the direct and indirect antibacterial properties for the short term, the sustained antibacterial ability is also important for orthopedic/dental implants (Guo et al., 2021). To assess the sustained antibacterial ability, the antibacterial properties of pDA@NP-modified samples was tested for 1, 3, and 5 days by serial dilution method as shown in Supplementary Figures S2A–C. The results demonstrate that with the increases of bacterial inoculation amount and culture time, the pH 4 samples gradually lost the ability to inhibit surrounding bacteria, and only the pH 4-H samples still inhibited bacterial proliferation and maintained a clear inoculum on the day 5. To test the stability of these pDA@NP-modified samples, samples from the same batch as above were immersed in physiological saline for 7 days, and then the spread plate method (Supplementary Figure S1D) and the turbidimetric method were used to evaluate their antibacterial ability (Supplementary Figure S3) (Assenza et al., 2012). The results demonstrate that only pH 4-H samples could inhibit bacterial proliferation while others did not show obvious antibacterial activity. To test the continued antibacterial property of samples of pH 4-H, they were inoculated with *S.aureus* for fifth times, and as the results in Supplementary Figure S2D show, they could keep combatting bacteria. All these results indicated that the pH 4 and pH 4-H samples had a good ability to inhibit the proliferation of gram-positive and gram-negative bacteria around them in the short term, but the pH 4-H sample had a stronger and more durable bacterial inhibition ability due to the release of a larger amount of silver (Figures 2B,C). The pH 7-H and pH 10-H surfaces released large amounts of silver but showed no antibacterial activity, which may indicate that silver was released by particle stripping and that pDA will function as a shell even when particles stripped from the surface as a whole.

### *In Vivo* antibacterial properties

**FIGURE 4:**
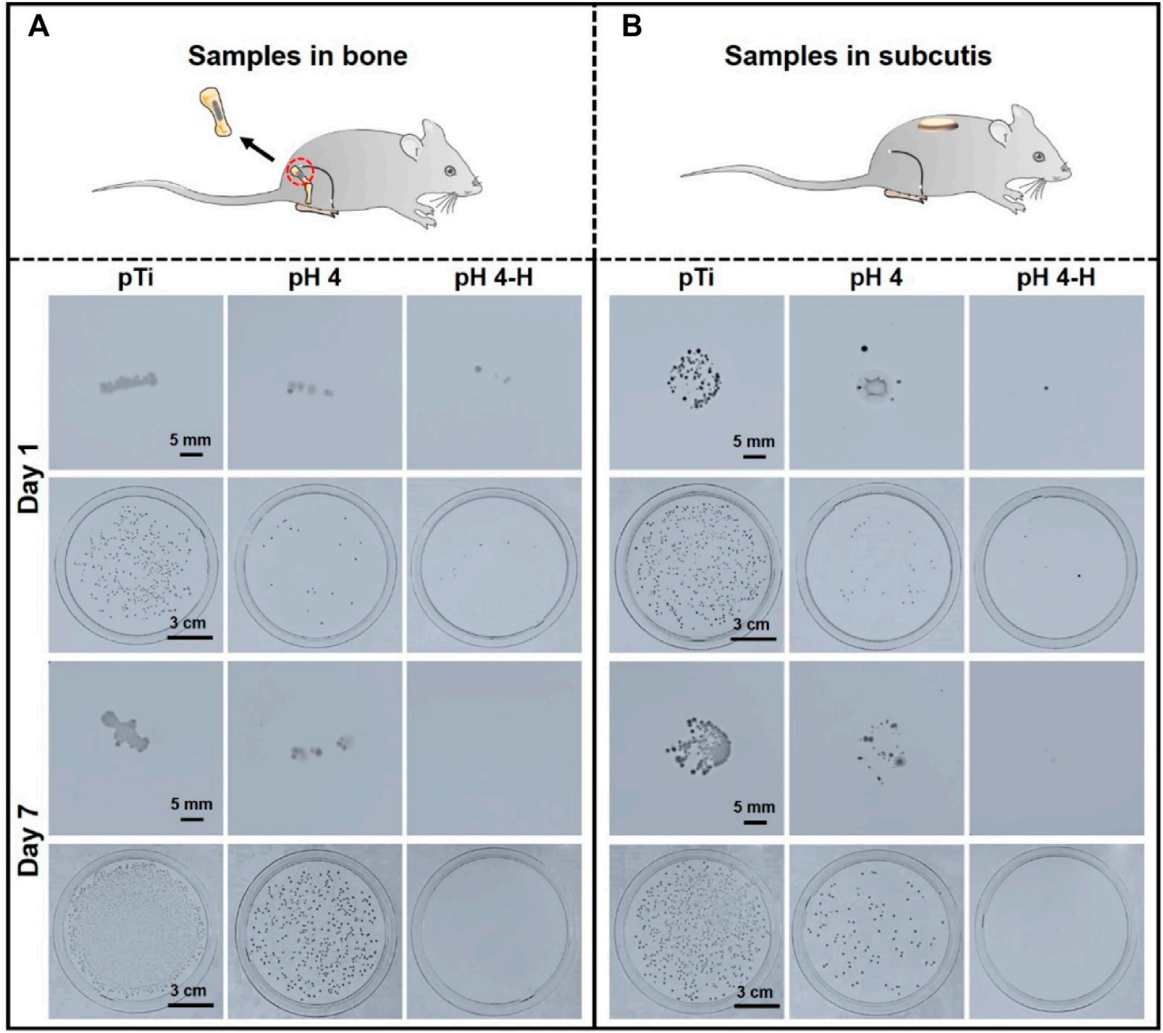
*In-vivo* antibacterial assay of the pH 4 and pH 4-H treatments:**(A)** Titanium rods dipped in bacteria solution were implanted into the femoral medullary cavity of rat models for one and 7 days; **(B)** titanium discs dipped in bacteria solution were implanted into the subcutaneous location of rat models for one and 7 days.

More than 700 bacterial species inhabit the oral cavity, so the influence of bacteria cannot be avoided (Chattopadhyay et al., 2019). Bacterial infection will continue to develop if invasion happens, so the *in vivo* antibacterial ability needs to be evaluated. Herein, a rat model was developed to investigate the antibacterial ability of these AgNP-loaded coatings. Since implants will contact both bone and soft tissue, titanium rods and titanium discs dipped in bacteria solution were implanted into the femoral medullary cavity and a subcutaneous location of a rat model, respectively (Shimazaki et al., 2010; Masamoto et al., 2021). Samples of the pH 4 and pH 4-H treatments were chosen for further evaluation because only the samples prepared in acid conditions presented good antibacterial ability in the *in vitro* experiment. After 1 and 7 days of implantation, the samples were removed and rolled on agar to be recultured for 24 h. As shown in Figure 4A, after 1 day of implantation, a large number of bacteria adhered to the pure titanium rod surface, and the number of bacteria that adhered to the pH 4 and pH 4-H surfaces was significantly lower than that of the pure titanium rod surface. In addition, the bacteria adhered to the samples were detached by ultrasound for the spread plate test. The results showed that the number of bacterial colonies on pTi, pH 4 and pH 4-H decreased successively, and only sporadic colonies were found on the surfaces of pH 4 and pH 4-H, which was consistent with the results of the rolling culture. After 7 days of implantation, the results of the rolling culture and spread plate method showed that the number of adherent bacteria on the surface of pTi and pH 4 increased significantly compared with that of 1 day culture, and the number of bacteria on the pH 4 surface was still much lower than that on the pTi surface. These results indicated that the pH 4-H surface exhibited better *in vivo* antibacterial properties. Titanium discs inoculated with bacteria were subcutaneously implanted into rats. After 1 and 7 days of culture, the samples were removed and pressed on an agar culture plate; at the same time, bacteria were detached by ultrasound for the spread plate test. As shown in Figure 4B, the number of bacteria adhered to the surface of pTi increased with the extension of implantation time, showing no antibacterial ability. The pH 4 surface showed antibacterial ability at the initial stage of implantation, but with prolonged time, the number of bacteria on the surface increased, indicating that the pH 4 sample could only delay bacterial proliferation. In contrast, pH 4-H showed stronger antibacterial ability with the extension of implantation time, and no living bacteria were found on the surface of the implant after 7 days due to the high silver loading and the relatively abundant Ag release, which formed an antibacterial area around the implant. These results indicated that the pH 4-H surface possessed better *in vivo* antibacterial properties at the gap between samples with bone or soft tissue.

### Biocompatibility of AgNPs loaded coatings

**FIGURE 5:**
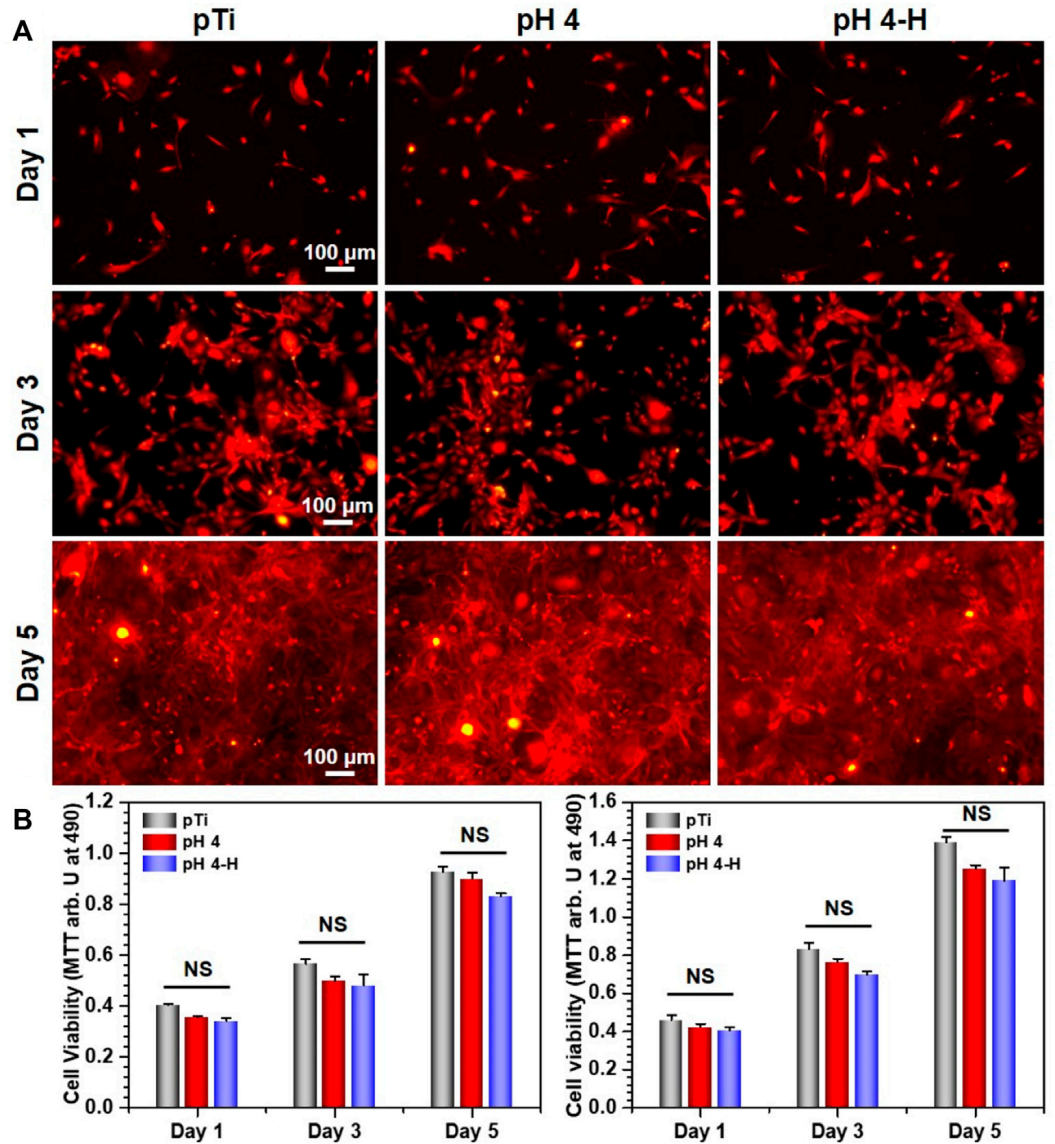
Biocompatibility evaluation of samples in group pH 4 and pH 4-H:**(A)** YF555-phalloidin (for F-actin) and DAPI (for nuclei) staining of MC3T3-E1 on various samples; **(B)** Left: MTT tests for MC3T3-E1of various samples. Right: MTT tests for MC3T3-E1 of leach liquor of various samples. NS: no significance.

**FIGURE 6:**
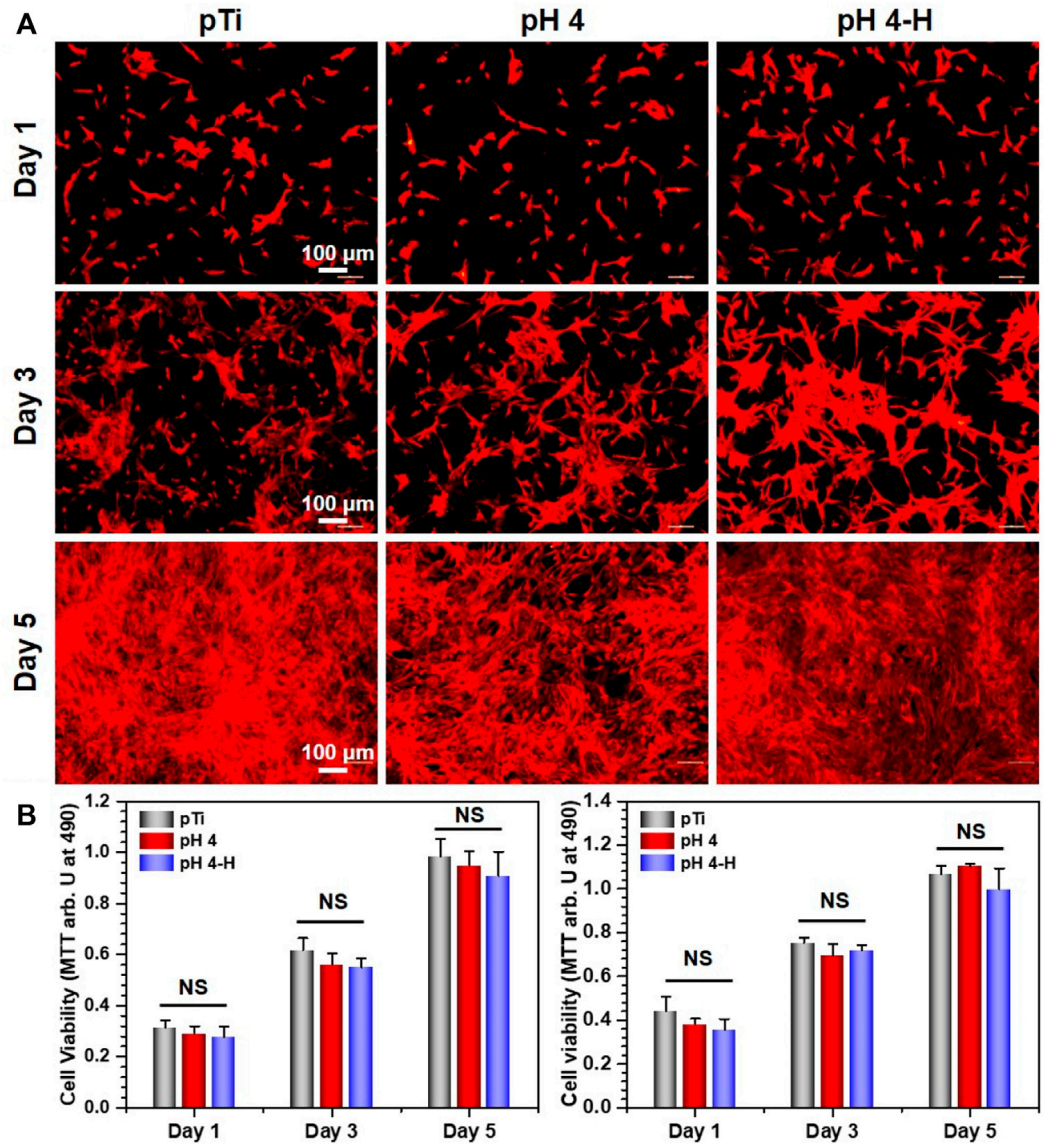
Biocompatibility of the pH 4 and pH 4-H treatments: **(A)** YF555-phalloidin (for F-actin) and DAPI (for nuclei) staining of NIH-3T3 on various samples; **(B)** Left: MTT tests for NIH-3T3 of various samples. Right: MTT tests for NIH-3T3 of leach liquor of various samples. NS: no significance.

Surface antibacterial modification is considered to be a short-term method because the antibacterial substance on the surface is gradually embedded or released, leading to a decline in antibacterial ability. To ensure a long-term therapeutic effect, it is very important to achieve rapid osseointegration with alveolar bone at the bone-implant interface and soft tissue integration at the trans-mucosal region to seal the crevice between the implant and surrounding tissues to prevent bacterial invasion (Guo et al., 2021). Therefore, cell fluorescence staining and MTT assays were used to evaluate the biocompatibility of the samples with the mouse osteoblastic cell line (MC3T3-E1) (Figure 5) and the mouse fibroblastic cell line (NIH-3T3) (Figure 6). As shown in Figure 5A and Figure 6A, the results of fluorescence staining showed that the number of osteoblasts and fibroblasts attached to pH 4 and pH 4-H was similar to that of the pTi. After 5 days of culture, all surfaces were completely covered by osteoblasts and fibroblasts. In addition, to assess the toxicity to the surrounding tissue, a 1-day extraction of the samples were collected to assess their toxicity by crystal violet staining and MTT tests. As the results of Supplementary Figure S5 show, there was also no obvious difference in the number of osteoblasts and fibroblasts among the leach liquor of various samples. These results suggest that samples of pH 4-H did not show significant cytotoxicity. The MTT assay (Figure 5B and Figure 6B) showed that the activity of osteoblasts and fibroblasts on the surface or in the leach liquor of pTi, pH 4 and pH 4-H samples decreased successively. Compared to that of pTi, the relative growth rate (RGR) of pH 4-H after 5 days was 89.5% for osteoblasts and 92.4% for fibroblasts, and in the leach liquor, the it was 85.6% for osteoblasts and 93.4% for fibroblasts, indicating that the pH 4-H sample had low cytotoxicity and was slightly more toxic to osteoblasts than to fibroblasts. In our previous study, silver-loaded composite coatings were prepared by depositing dopamine and then silver nitrate in an alkaline environment. When the concentration of silver nitrate was 10 mg/mL (Chen et al., 2016) and 0.1 mg/mL (Wang et al., 2021b), the RGRs of osteoblasts on these surfaces at 5 days was 36.7% and 79.2%, respectively. In this study, the pH 4 and pH 4-H surfaces demonstrated remarkable superiority, suggesting that the silver in the previous study was completely exposed on the surface, but here it was deposited in the manner of pDA@NP complex, and the pDA coating outside the complex reduced the exposure of silver on the surface and the amount of silver released, thus reducing the direct cytotoxicity of AgNPs.

**FIGURE 7:**
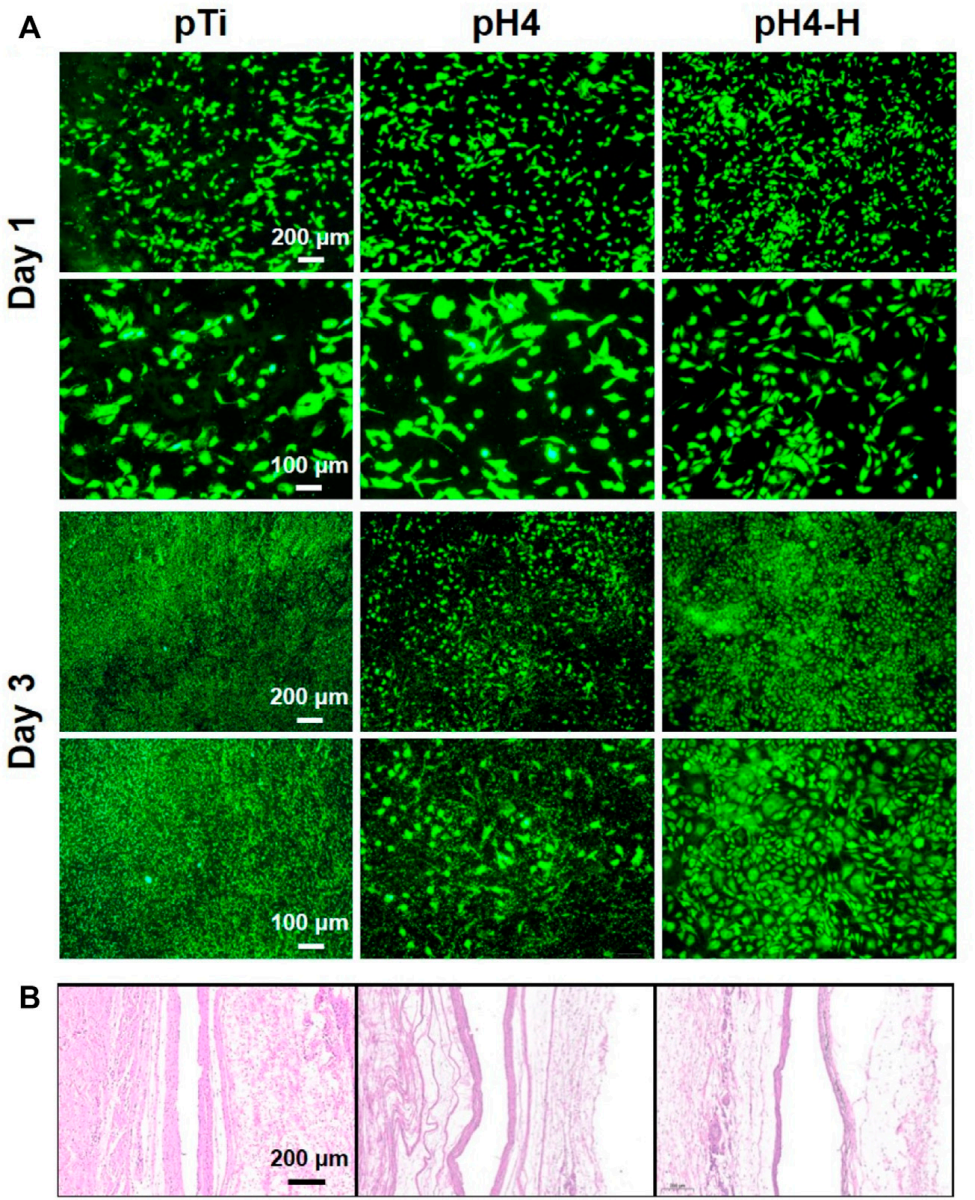
Co-culture of bacterial and cell or tissue: **(A)** Osteoblasts (MC3T3-E1) were seeded on different samples inoculated with S. aureus and cultured for 1 and 3 days; **(B)** HE staining about local tissue (pretreated with S.aureus) of various samples after 1 month of subcutaneous implantation.

Since the oral cavity is rich in bacteria, dental implants operate where host cells and bacteria co-exist and the competition is intense (Fernández et al., 2011). Here, the coculture of bacteria with cells was performed to evaluate the biological effect of antibacterial implants in a complex environment (Wang et al., 2016). Specifically, osteoblasts (Figure 7) and fibroblasts (Supplementary Figure S6) were seeded on samples inoculated with *S. aureus* and cultured for 1 and 3 days. After 1 day of culture, many osteoblasts were observed on both the pTi surface and the modified surface, while there were a few bacteria on pTi and pH4. After 3 days of culture, many live bacteria and sporadic apoptotic cells were observed on the pTi surface, and many adherent bacteria covered the pH 4 surface, while there were a large number of healthy osteoblasts and fibroblasts on the pH 4-H surface. The results were consistent with the antibacterial evaluation (Figure 3 and Supplementary Figure S1D, Supplementary Figures S2, S3), which showed that the pH-4 sample possessed limited antibacterial ability and could only delay bacterial proliferation, while the pH 4-H sample had sufficient capacity to inhibit the proliferation of bacteria. Thus, we speculated that if the surface cannot completely inhibit or kill bacteria, bacteria can ultimately thrive in the gap between implant and tissue, which will also lead to implant failure, so the pDA@NP complex prepared in acidic conditions that possess strong antibacterial ability are indispensable. Clinically, the service sites of orthopedic and dental implants are not only bone but also soft tissue, and soft tissue is more easily infected by bacteria and causes postoperative failure ^11^. Therefore, specimens pretreated with bacteria were implanted into the subcutis to evaluate the subcutaneous antibacterial properties. After 1 month of implantation, the tissue around the materials was harvested, and H&E staining for histological examination was carried out, the results of which are shown in Figure 7B. As a representation of soft tissue inflammation, the fibrous capsule thickness around pTi, pH 4 and pH 4-H was 80–90, 60–70, and 30–40 μm, respectively, indicating that the pH 4-H surface had a stronger inhibitory effect to microorganisms, which reduced microbe-related inflammation, thus preventing capsule formation. Implantation-induced fibrotic capsules are considered to be caused by a tissue response, so the results also demonstrate that these Ag-loaded surfaces would not cause inflammation. Instead, the surfaces with durable antibacterial properties could inhibit the tissue response caused by bacteria, which makes them promising candidates for orthopedic and dental implants.

**FIGURE 8:**
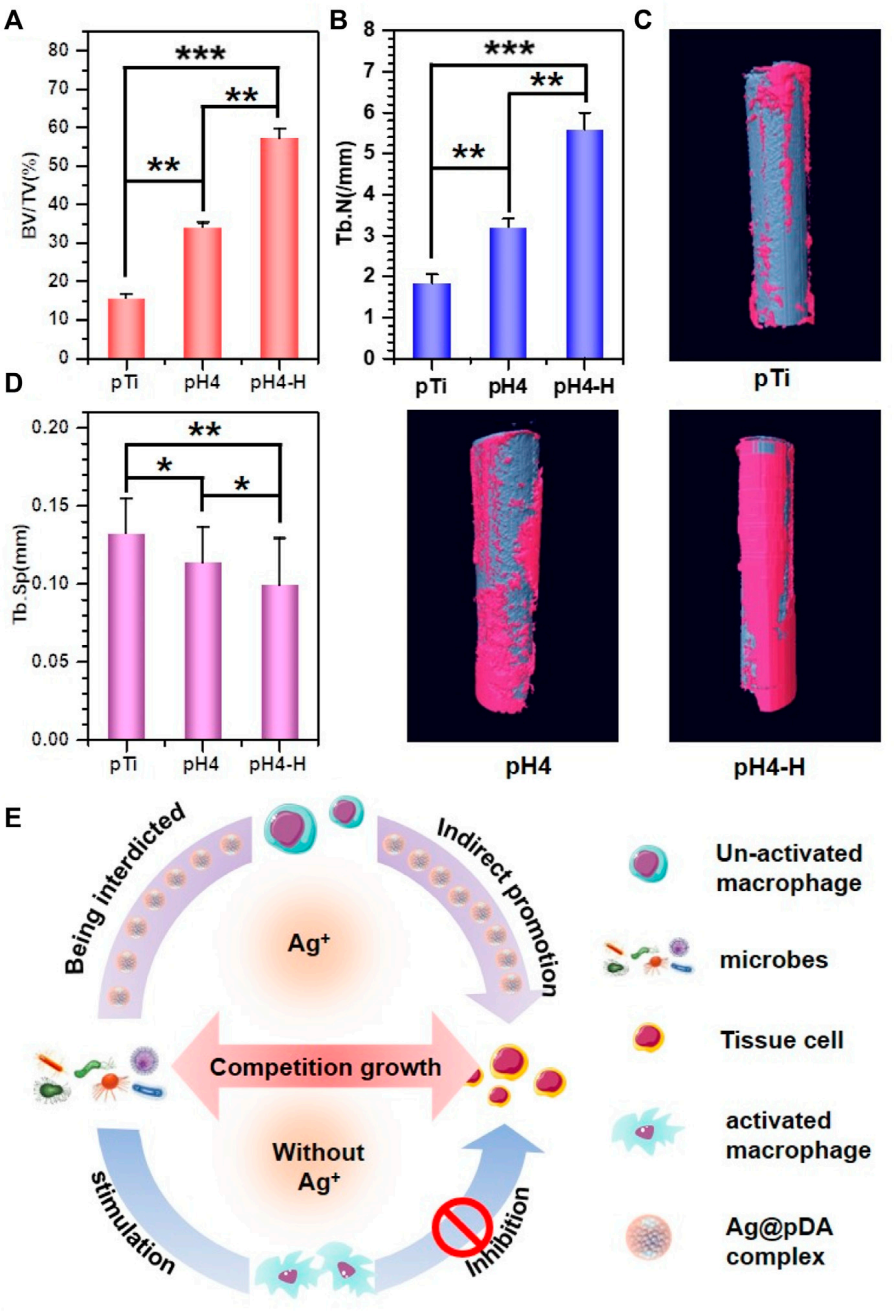
3D micro-CT modeling of various samples: **(A–C)** Quantitative evaluation of new bone surrounding the implants on the basis of the percentage of bone volume to tissue volume (BV/TV) **(A)**, Tb.N **(B)** and Tb.SP **(C)** value. **(D)** 3D micro-CT reconstructed images; **(E)** schematic illustration of induced-osteogenesis hypothesis. Data were expressed as the mean ± SD, *n* = 3. Statistically significant differences are indicated by *p < 0.05, **p < 0.01 or ***p < 0.001.

Recent studies have demonstrated the excellent reproducibility and accuracy of micro-CT measurements of bone morphology (Bouxsein et al., 2010; Liu et al., 2021). Therefore, 3D micro-CT analysis was performed to investigate bone regeneration under bacterial infection conditions (Figure 8D). Prior to implantation, *S. aureus* inoculum was added, and then Ti rods were implanted into the infected marrow cavity (Ribeiro et al., 2012). According to the results of Figures 8A–C), the Ti rod with pH 4 heat treatment-modified AgNPs presented the highest percentage of bone volume to tissue volume (BV/TV), which may be due to the strongest antibacterial properties of this sample. Moreover, consistent with the BV/TV results, the increased trabecular number (Tb. N) and lower trabecular separation (Tb. SP) also indicate that pH 4 heat treatment-modified AgNPs expressed exceptional osteogenesis capacity. In contrast, severe bone loss occurred around the pTi, pH 4-modified Ti rods due to their insufficient antibacterial properties. Bone regeneration is a complex process (Figure 8E), during which tissue cells, immune cells, microbes and implants interact with each other (Trindade et al., 2016). Apart from host cells, microbes and implants are two main types of foreign matter. Pathogenic bacteria will compete with local tissue cells and their secretion will inevitably influence the normal physiological behavior of the host cells. Furthermore, once bacteria-triggered dysbacteriosis occurs, immune cells are activated and a series of inflammatory reactions, such as inflammatory factors (IL-6, IL-8, TNF-α, etc.) secretion, tissue fluid exudation, vascular permeability increase and blood cell exudation, are initiated, which ultimately prolong the tissue repair (Sherwood and Toliver-Kinsky, 2004). Therefore, implants with strong antibacterial ability will reduce the incidence rate of microbe-related inflammation thus promoting bone regeneration, so, pH 4-H exhibits better osteogenic capability due to the excellent release manner, the high antibacterial ability and appropriate cytocompatibility. The heat treatment improved the oxidative crosslinking of dopamine, thus enhancing the stability of the pDA@NP complex. The residual dopamine and Ag^+^ after simple washing could maintain the pDA@NP content at a high level, and pH plays a key role in determining the Ag enwrapping manner, which ultimately realize the balance of antibacterial and cytotoxicity.

## Conclusion

A pDA@NP complex was successfully prepared on porous Ti by a redox reaction of silver with dopamine under acidic conditions. Acidic conditions and heat treatment are two key factors to ensure enough Ag content and proper release manner which endow the surface with antibacterial properties and excellent cell compatibility. Surface morphology, XPS and ICP-MS synthetically proved that pDA coatings were formed around AgNPs and different enwrapping behavior was exhibited. Two kinds of bacteria: *S. aureus* and *Aa* were used to detect the bacterial properties, and two kinds of cell, MC 3T3-E1 and MC 3T3 fibroblasts, were chosen to investigate their cell compatibility. Both the *in vitro* and *in vivo* experiments demonstrate that the pDA@Ag complex formed at pH 4 and 150°C showed the strongest antibacterial property and appropriate cytotoxicity. Microbe-related infection models were build and the tissue response in bacterial-exposed environment proved that pH4-H could prevent the microbe-related inflammation, thus promoting the tissue repair indirectly.

## Data Availability

The original contributions presented in the study are included in the article/Supplementary Material, further inquiries can be directed to the corresponding authors.
