# Supplementary material for: Controllable AgNPs encapsulation to construct biocompatible and antibacterial titanium implant
Source: Front Bioeng Biotechnol. 2022 Nov 30;10:1056419. doi: 10.3389/fbioe.2022.1056419 (PMC9747934; doi:10.3389/fbioe.2022.1056419)
Supplement: Supplementary file 1 [file DataSheet1.PDF]

Supporting Information

Controllable AgNPs encapsulation to construct biocompatible and antibacterial titanium implant

Zhangao Wei [†, ⊤], Kexin Li[†, ⊤], Shuang Wang [†, ⊤], Lan Wen [‡], Linghan Xu[†], Yankai Wang [†], Zirui Chen[†], Wei Li [†], Hua Qiu[†, *], Xiangyang Li[†, *], Jialong Chen[†, *]

[†]Stomatologic Hospital and College, Anhui Medical University, Key Laboratory of Oral Diseases Research of Anhui Province, Hefei, Anhui 230032, China

[‡]Department of Neurosurgery, West China Hospital, Sichuan University, Chengdu, China

China. E-mail addresses:

qiuhua1990@foxmail.com (Hua Qiu),

hlxiangyang@163.com (Xiangyang Li),

jialong_dt@126.com (Jialong Chen)

List of Contents

FIGURE S1. Assessment of antibacterial activity of different samples: 2

FIGURE S2. Assessment the sustained antibacterial ability: 3

FIGURE S3. Bacterial amount in the inoculum of various samples soaked in physiological saline for 7 days.. 4

FIGURE S4. XPS full-spectrum.. 5

FIGURE S5. Assessment of the toxicity to the surrounding tissue 6

FIGURE S6. Co-culture of bacterial and cell or tissue....................................... 7

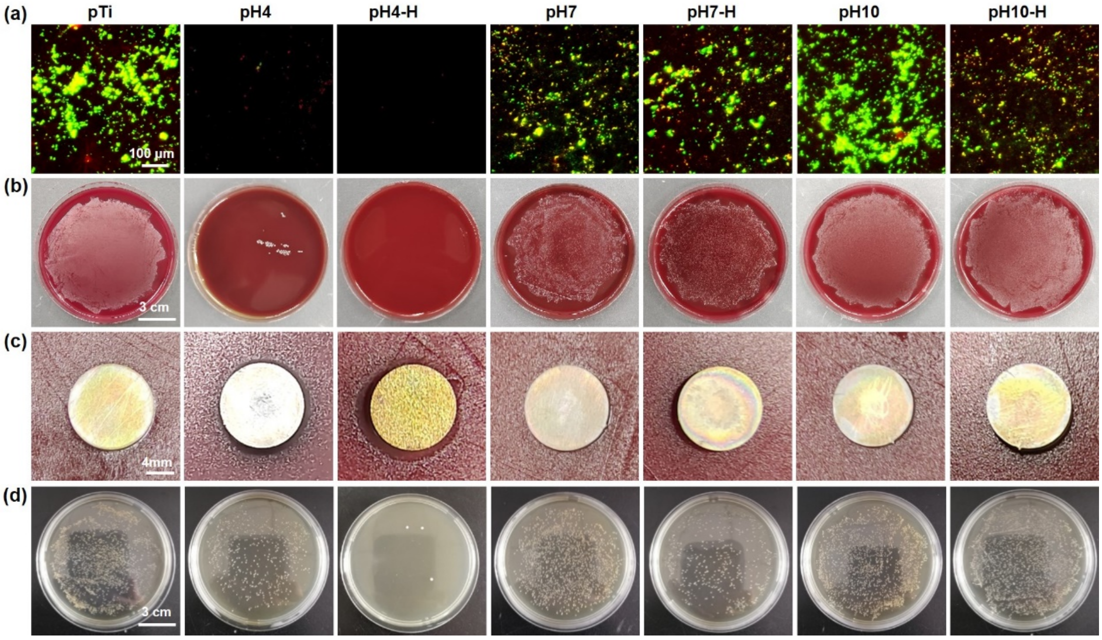

FIGURE S1. Assessment of antibacterial activity of different samples: (a) Live/Dead bacteria staining of Aa on various samples. (b) Antibacterial property *(Aa)* of various sample surfaces was determined by spread plate method. (c) The diameter of inhibition zone around various samples for *Aa*. (d) Bacterial amount of various sample surfaces soaked in physiological saline for 7 days followed by incubation with *S. aureus* for 24h was determined by spread plate method.

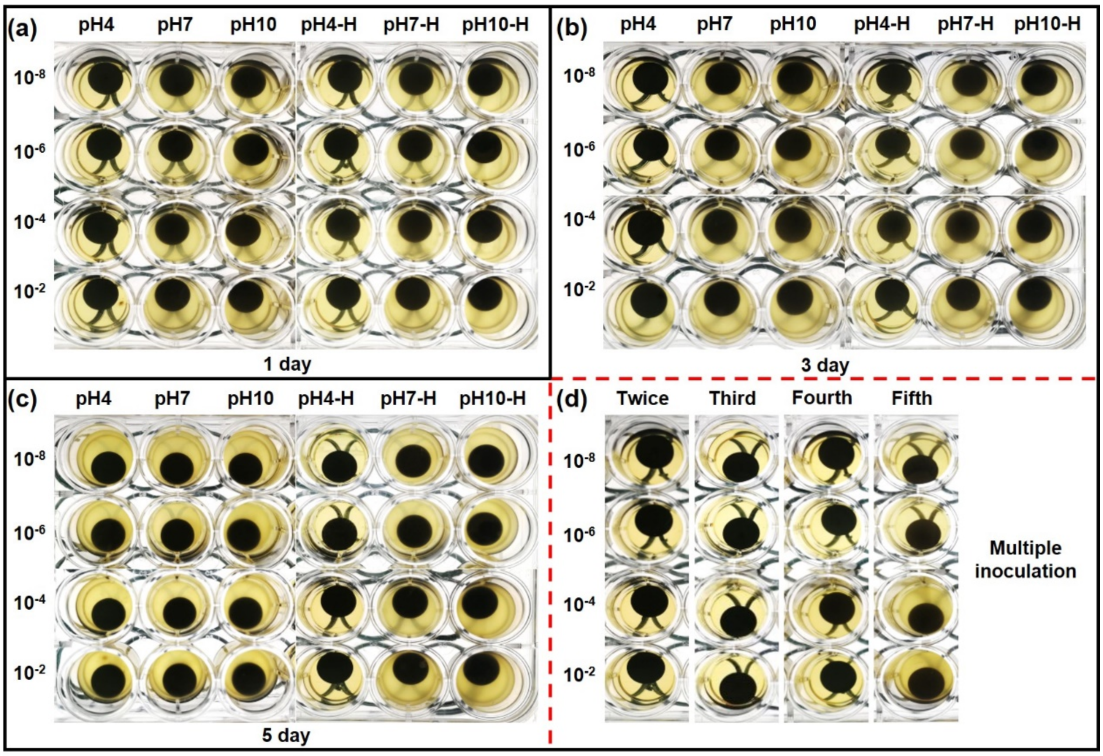

FIGURE S2. Assessment the sustained antibacterial ability: To assess the sustained antibacterial ability, the dopamine/Ag-modified samples was tested for 1 (a), 3 (b) and 5 days (c) by serial dilution method. (d) pH 4-H were multiple inoculated (from twice to fifth) with *S. aureus*.

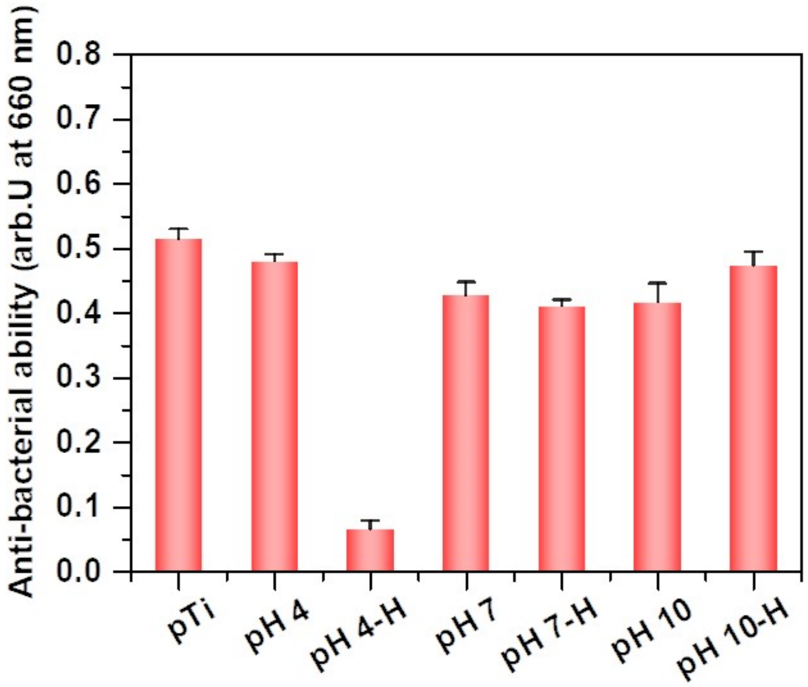

FIGURE S3. Bacterial amount in the inoculum of various samples soaked in physiological saline for 7 days followed by incubation with S. aureus for 24 h was determined by turbidimetric method.

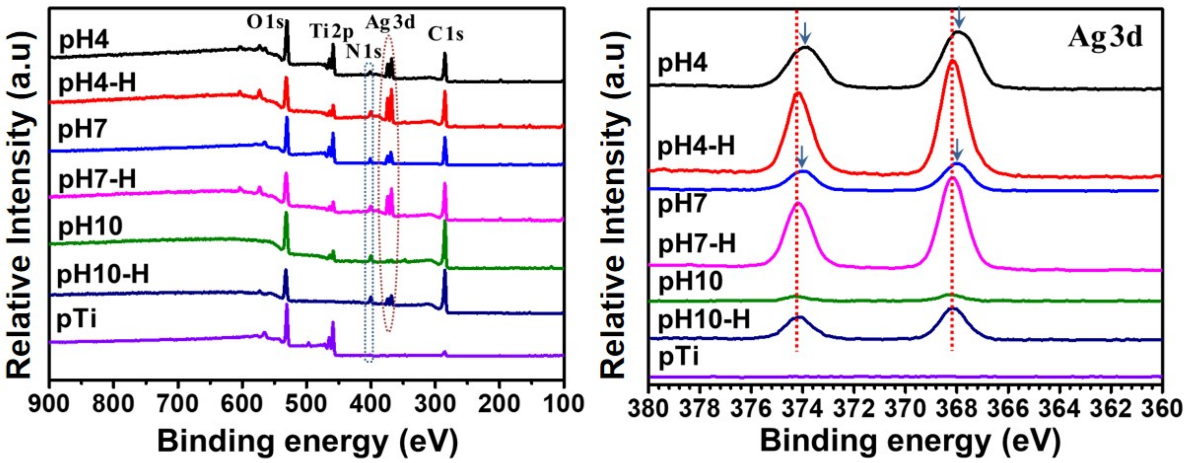

FIGURE S4. XPS full-spectrum analyses of samples.

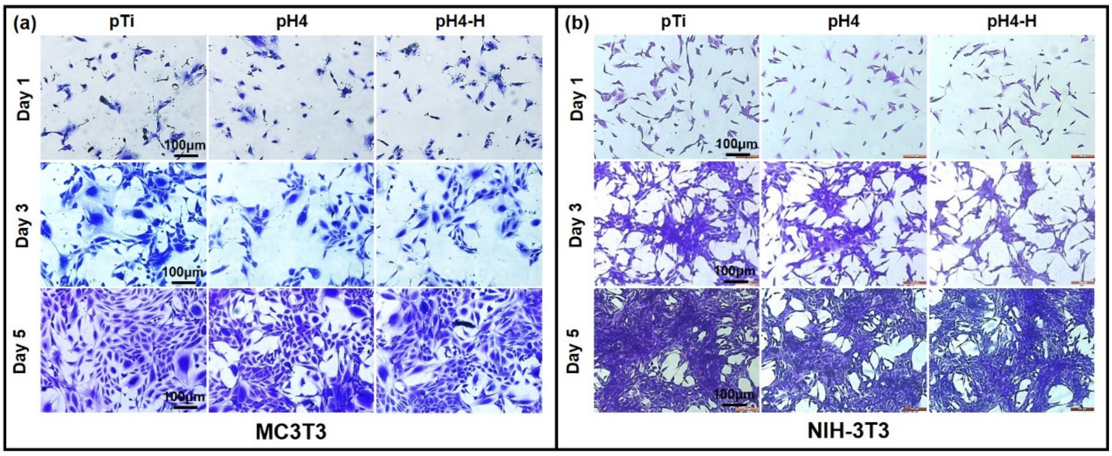

FIGURE S5. Assessment of the toxicity to the surrounding tissue by crystal violet staining of MC3T3-E1(a) and NIH-3T3(b).

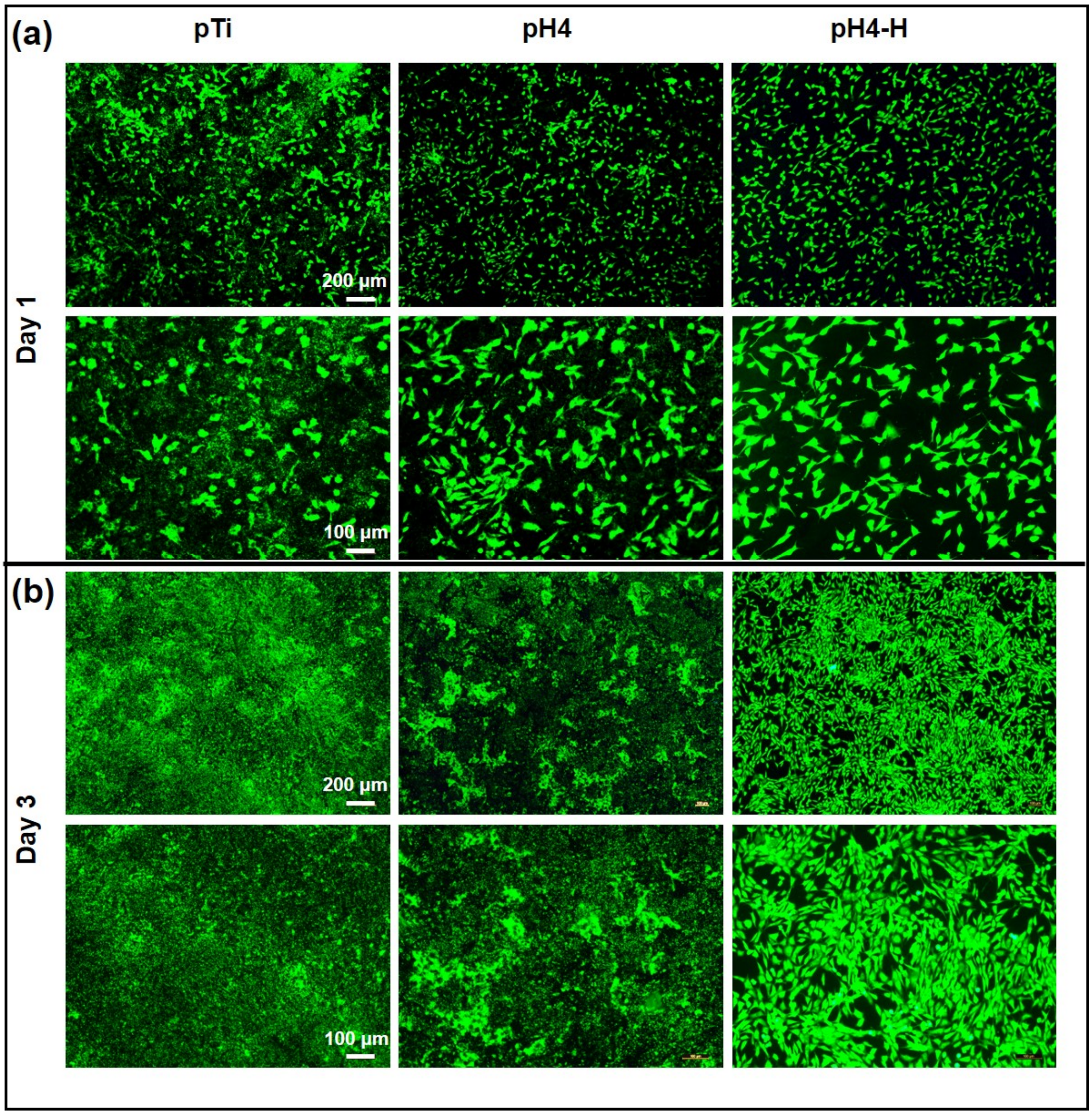

FIGURE S6. Co-culture of bacterial and cell or tissue. (a) Fibroblasts (NIH-3T3) were seeded on different samples inoculated with *S. aureus* and cultured for 1 day. (b) Fibroblasts (NIH-3T3) were seeded on different samples inoculated with *S. aureus* and cultured for 3 days.
